# Intolerance to Angiotensin Converting Enzyme Inhibitors in Asthma and the General Population: A UK Population-Based Cohort Study

**DOI:** 10.1016/j.jaip.2021.04.055

**Published:** 2021-09

**Authors:** Daniel R. Morales, Brian J. Lipworth, Peter T. Donnan, Huan Wang

**Affiliations:** a Division of Population Health and Genomics, University of Dundee, Dundee, United Kingdom; b Health Data Research (HDR)-UK; c Department of Public Health, University of Southern Denmark, Denmark; d Scottish Centre for Respiratory Research, University of Dundee, Dundee, United Kingdom; e Division of Population Health and Genomics, University of Dundee, Dundee, United Kingdom; f Dundee and Epidemiology Biostatistics Unit, University of Dundee, Dundee, United Kingdom

**Keywords:** Asthma, Angiotensin converting enzyme, Cough, Epidemiology, Hypertension, ACEI, Angiotensin converting enzyme inhibitor, AHR, Airway hyperresponsiveness, ARB, Angiotensin-II receptor blocker, BMI, Body mass index, BTS, British Thoracic Society treatment step, COPD, Chronic obstructive pulmonary disease, HR, Hazard ratio, ICS, Inhaled corticosteroid, NNT, Number needed to treat

## Abstract

**Background:**

Angiotensin converting enzyme inhibitor (ACEI) intolerance commonly occurs, requiring switching to an angiotensin-II receptor blocker (ARB). Angiotensin converting enzyme inhibitor intolerance may be mediated by bradykinin, potentially affecting airway hyperresponsiveness.

**Objective:**

To assess the risk for switching to ARBs in asthma.

**Methods:**

We conducted a new-user cohort study of ACEI initiators identified from electronic health records from the UK Clinical Practice Research Datalink. The risk for switching to ARBs in people with asthma or chronic obstructive pulmonary disease and the general population was compared. Adjusted hazard ratios (HRs) were calculated using Cox regression, stratified by British Thoracic Society (BTS) treatment step and ACEI type.

**Results:**

Of 642,336 new users of ACEI, 6.4% had active asthma. The hazard of switching to ARB was greater in people with asthma (HR = 1.16; 95% confidence interval [CI], 1.14-1.18; *P* ≤ .001) and highest in those at BTS step 3 or greater (HR = 1.35, 95% CI, 1.32-1.39; and HR = 1.18, 95% CI, 1.15-1.22, *P* ≤ .001 for patients aged ≥60 and <60 years, respectively). Hazard was highest with enalapril (HR = 1.25, 95% CI, 1.18-1.34, *P* ≤ .001; HR = 1.44, 95% CI, 1.32-1.58, *P* ≤ .001 for BTS step 3 or greater asthma). No increased hazard was observed in chronic obstructive pulmonary disease or those younger than age 60 years at BTS step 1/2. The number needed to treat varied by age, sex, and body mass index (BMI), ranging between 21 and 4, and was lowest in older women with a BMI of 25 or greater.

**Conclusions:**

People with active asthma are more likely to switch to ARBs after commencing ACEI therapy. The number needed to treat varies by age, sex, BMI, and BTS step. Angiotensin-II receptor blocker could potentially be considered first-line in people with asthma and in those with high-risk characteristics.

***What is already known about this topic?*** Many people are intolerant to angiotensin converting enzyme inhibitors owing to cough and require switching to an angiotensin-II receptor blocker. Angiotensin converting enzyme inhibitors may affect airway hyperresponsiveness in asthma, possibly mediated by bradykinin or cough reflex sensitivity.

***What does this article add to our knowledge?*** People with asthma are generally at increased risk for switching to angiotensin-II receptor blockers from angiotensin converting enzyme inhibitor therapy, and the risk is greatest in those with more severe asthma. The absolute risk for switching varies by age, sex, and body mass index.

***How does this study impact current management guidelines?*** Angiotensin-II receptor blockers could be considered first-line in older people with asthma or young people with more severe asthma, including those with other high-risk characteristics.

## Introduction

Asthma is a highly prevalent disease causing significant morbidity, mortality, and health care cost.^1^ Comorbidity in asthma is common; 62.6% of people with asthma were reported to have one or more comorbidities, and the likelihood of having coronary artery disease, congestive heart failure, peripheral vascular disease, cerebrovascular disease, hypertension, diabetes, and chronic kidney disease is significantly greater in people with asthma compared with the general population.^2^^,^^3^ Angiotensin-converting enzyme inhibitors (ACEIs) are commonly prescribed medicines indicated for the management of these chronic diseases.^4^ Angiotensin-converting enzyme inhibitors block the enzyme responsible for converting the peptide hormone angiotensin-I to angiotensin-II, which stimulates aldosterone release and causes vasoconstriction. Although ACEIs have beneficial effects in managing these chronic diseases, many patients are intolerant of long-term ACEIs. The most common reason is a dry persistent cough. This adverse drug reaction is thought to occur in around 10% of people treated with ACEI and may be related to increased levels of bradykinin.^5^ This adverse reaction is considered a class effect of ACEI, which suggests that even low doses may also alter bradykinin levels in susceptible patients.

In people who develop ACEI intolerance from cough, it is recommended that patients are switched to angiotensin-II receptor blocker (ARB) therapy.^5^ Angiotensin-II receptor blockers have properties similar to those of ACEIs but do not cause a persistent dry cough. They inhibit angiotensin-II in a highly selective manner through a mechanism that does not alter bradykinin levels. However, irrespective of the cause, having to switch treatments increases health care resource use, treatment burden, and treatment disutility, and may delay establishing effective preventative therapy for the underlying indication. Despite being an important health economic factor, many drug formularies and guidelines still recommend first-line treatment with ACEIs, usually because of cost.^6^

A key tenet in the pathogenesis of asthma is airway hyperresponsiveness (AHR), which can be affected by a variety of environmental stimuli.^7^^,^^8^ Bradykinin is a proinflammatory mediator that can cause bronchoconstriction and lung inflammation.^9^ It is therefore plausible that treatment with ACEIs may exacerbate asthma symptoms through bradykinin accumulation, leading to worsening AHR, which may in turn increase the incidence of cough and switching to ARBs.^10^ However, there is limited evidence regarding the effect of ACEI exposure in patients with asthma. The aims of this study were to (1) examine ACEI drug use in people with asthma, (2) assess the association of switching to ARBs in people with asthma compared with the general population, and 3) characterize patients at greater risk.

## Methods

### Data source

The UK Clinical Practice Research Datalink (CPRD) GOLD database was used to identify a large UK cohort of people with active asthma. Clinical Practice Research Datalink GOLD contains anonymized electronic medical records from more than 680 general practices covering more than five million people in the United Kingdom with linked health data about patient demographics, prescriptions, diagnoses, hospitalizations, and deaths. Patients are broadly representative of the UK general population in terms of age, sex, and ethnicity.^11^ General practices and patients within CPRD GOLD are required to meet defined quality standards to contribute data; diagnoses have high validity, including for asthma that has a positive predictive value for respiratory disease of around 90%.^12^^,^^13^ It has also been deemed to meet regulatory requirements to be used in a regulatory context.^14^

### Study cohort

An open cohort of adults aged 18 years and over was identified from January 1, 1998 through June 30, 2014. This period reflects the start of database availability and the latest data available at the time of data extraction. Patients were required to be registered with a general practice providing up-to-standard data for at least 1 year before cohort entry. The population was divided into patients with active asthma; the remainder formed the rest of the general population. People with active asthma were defined using a validated code list for asthma and the receipt of at least two asthma medications with cohort follow-up commencing at the latest of these dates.^13^ Asthma medicines were defined by the use of inhaled short-acting β_2_-agonists, inhaled corticosteroids (ICS), inhaled long-acting β_2_-agonists, oral leukotriene antagonists, and oral methylxanthines.^15^ To reduce the chance of misclassification, people with a diagnostic code for asthma, who also had a diagnostic code for chronic obstructive pulmonary disease (COPD), interstitial lung disease, or bronchiectasis, were excluded from the active asthma population. For examining drug use, cohort exit (which results in right censoring) for all patients was defined as the earliest end of study period, deregistration from the general practice, or date of last data collection from the general practice, or death. For the analysis examining the risk for switching to an ARB after ACEI initiation, cohort entry was also defined by the date of the incident ACEI prescription in people with no prior ACEI or ARB exposure and cohort exit was also defined by the date of switching to an ARB or 180 days after ACEI discontinuation if no ARB had been initiated. For the switching analysis, patients prescribed an ARB on or before the incident ACEI were excluded. To test the robustness of the potential mechanism relating to asthma, we also examined this association in patients with COPD who acted as a negative control population. Patients with COPD are expected to be unaffected by the underlying pathophysiologic hypothesis targeting AHR and were identified also using a validated code list.^16^

### Exposures

**Figure E1:**
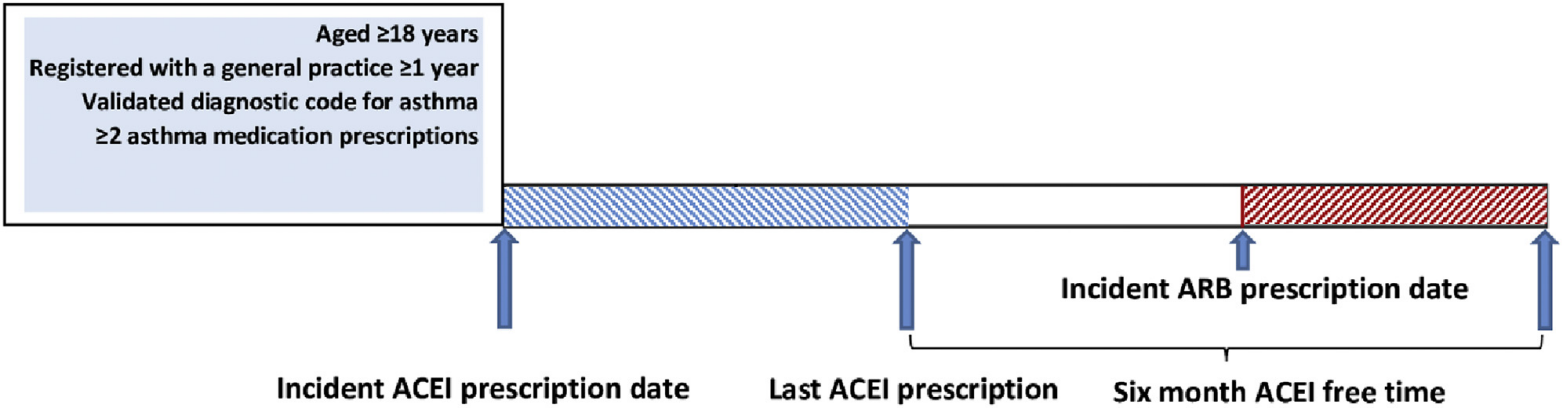
Exposure windows used to define switching to angiotensin-II receptor blocker (ARB) therapy after initiation of Angiotensin converting enzyme inhibitor (ACEI) therapy.

All ACEI and ARB prescriptions were identified for patients within the cohort. The date of incident ACEI therapy was defined as the first ever ACEI prescription occurring during cohort follow-up with no previous prescription at any point before this time. Angiotensin converting enzyme inhibitor discontinuation was defined by the date of an ACEI prescription with no further ACEI prescription after at least 6 months of this date. Switching to an ARB was defined by an incident ARB prescription issued within 6 months of the ACEI discontinuation date, with the date of the ACEI discontinuation representing day 1 of this 6-month period of follow-up (see Figure E1 in this article's Online Repository at www.jaci-inpractice.org). The list of ACEI and ARB drug codes are provided in Table E1 in this article's Online Repository at www.jaci-inpractice.org. For people who switched, the maximal ACEI dose prescribed before switching was calculated. Angiotensin converting enzyme inhibitor doses were standardized using ramipril equivalent doses (see Table E2 in this article's Online Repository at www.jaci-inpractice.org).

### Outcomes

The primary outcome was the relative hazard of switching from ACEI to ARB therapy in people with active asthma compared with the general population; trends in ACEI initiation and switching to ARBs were reported over the study period among the active asthma population. Patients could switch at any point after initiating ACEI therapy provided they met the definition of switching and had not been censored owing to one of the cohort exit criteria.

### Analysis

Trends in the quarterly prevalence of ACEI and ARB initiation and discontinuation were calculated for the active asthma population. The start of each quarter was defined as January 1, April 1, July 1, and October 1. The quarterly prevalence was age-standardized using the European standard population.^17^ The cohort analysis used Cox proportional hazards regression to calculate hazard ratios (HRs) for switching to an ARB after initiating ACEI therapy in people with asthma compared with the general population. Time in this time to event analysis was the difference in days between the date of the incident ACEI prescription and switching to an ARB or another cohort exit censoring event, as described earlier. Routine checks of the proportional hazards assumption were conducted by examining log-log plots. We used the entire population available to use within the database that met our criteria. Based on a two-group survival analysis, this cohort had 90% at α = 0.01 to detect a difference in relative hazard of 1.05 or greater. The Cox model was adjusted for the baseline confounders of age, sex, practice-level socioeconomic deprivation applied to the individual (defined by the Index of Multiple Deprivation categorized into quintiles), smoking status (categorized into smoker, ex-smoker, and nonsmoker); body mass index (BMI) (categorized into <20, 20 to 24, and ≥25), history of cardiovascular disease, and history of hypertension. We selected variables based on a search in the literature, known differences in characteristics of asthma patients, and indications for ACEI. A full model was fitted using all variables as main effects. The active asthma cohort was categorized into three groups according to baseline British Thoracic Society (BTS) asthma treatment step (1, 2 and ≥3), defined by prescribed asthma medication as a potential marker of severity and included in the model.^1^ The cohort was stratified by the most frequently prescribed types of ACEI and analyzed separately. Multiple imputation was used to impute missing data on BMI, deprivation and smoking status. The imputation model included all variables relating to clinical characteristics, medication exposure, and switching events. Multiple imputation used fully conditional specification, with linear regression for continuous variables and logistic regression for categorical variables with five imputations analyzed using Rubin's rules.^18^ We performed a complete case analysis to assess the impact of multiple imputation as a sensitivity analysis. To calculate an absolute measure, the rate of switching per 1000 patients was first calculated in the general cohort population and was then multiplied by the adjusted HR to calculate the expected number of switchers in asthma. The number of asthma patients needed to treat (NNT) with an ACEI for one person to switch to an ARB was then calculated by taking the reciprocal of this value. Data on absolute risk are presented stratified by age and sex, as done elsewhere.^19^^,^^20^

## Results

**Figure E2:**
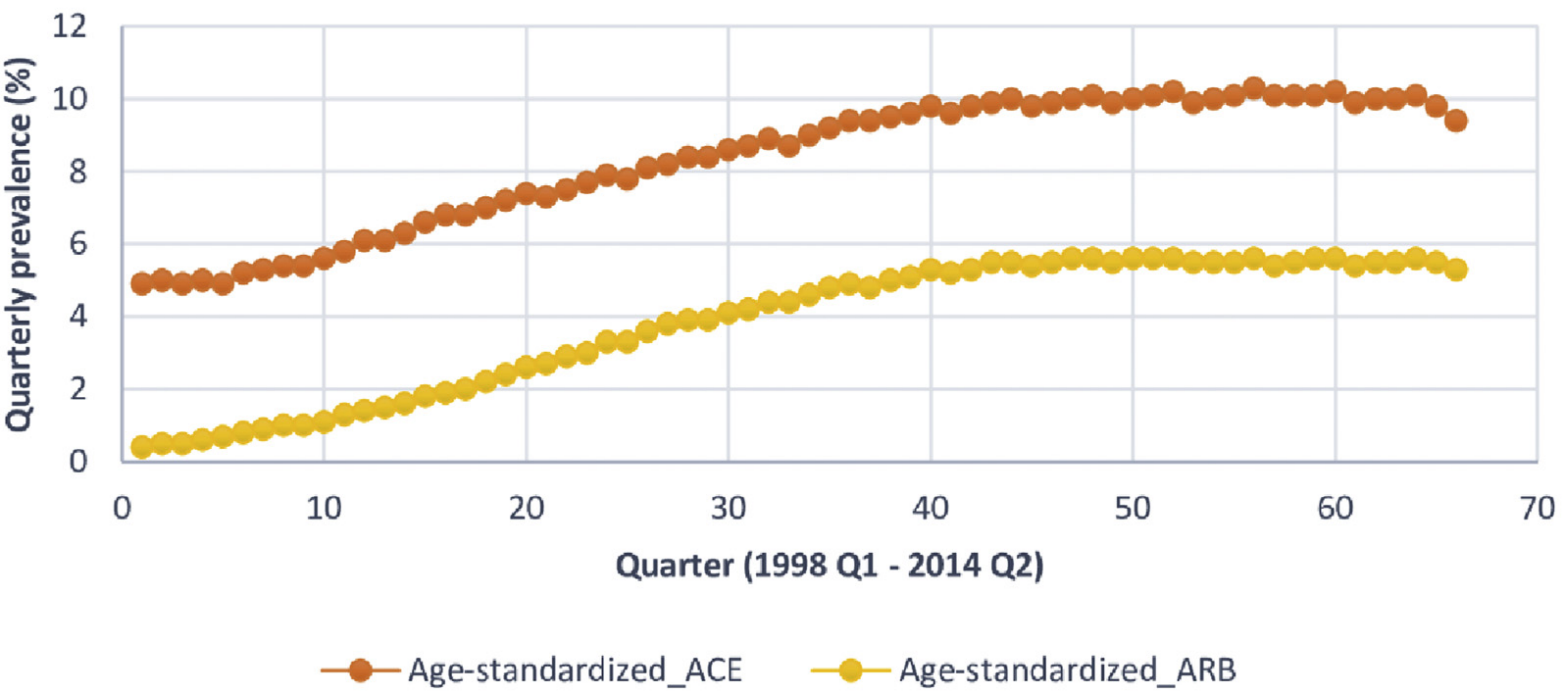
Age-standardized quarterly (Q) prevalence of angiotensin converting-enzyme inhibitor (ACE) inhibitors and angiotensin-II receptor blockers (ARBs) in patients with active asthma.

The active asthma cohort consisted of 521,857 adults (57.8% female; mean age, 39 years), of whom 66,895 patients were prescribed ACEIs (12.8%), 28,791 were prescribed ARBs (5.5%), and 16,203 were prescribed both (3.1%) individually at some point during the cohort follow-up. Trends in ACEI and ARB prescribing are presented in the Figure E2 (in this article's Online Repository at www.jaci-inpractice.org).

Among the entire population, 642,336 patients initiating ACEIs were identified, 40,953 of whom had active asthma (6.4%). The remainder formed the general population, 5.2% of whom had COPD. Table I lists patient characteristics. Fewer patients with active asthma were men or current smokers or had a history of cardiovascular disease. The most commonly prescribed ACEIs were ramipril, followed by lisinopril, perindopril, and enalapril. Overall, 17.4% of people with active asthma switched to an ARB after ACEI initiation, compared with 14.6% of the general population. Among those who switched, the number of general practitioner consultations and mean ramipril-equivalent doses before switching were similar between groups.

**Table I:** Demographic details and baseline covariates of people initiating ACEI therapy in the general population and in those with active asthma

Patient characteristics	Active asthma cohort (n = 40,953)	General population (n = 601,383)
Mean age, (SD)	58.7 (13.3)	64.4 (13.8)
Male sex (%)	17,274 (42.2)	315,463 (52.5)
Mean follow-up, y (SD)	3.0 (3.3)	3.3 (3.4)
Mean body mass index at baseline, kg/m^2^ (SD)	30.7 (6.7)	28.7 (5.9)
Missing body mass index, kg/m^2^ (%)	1314 (3.2)	39,519 (6.6)
Practice level deprivation (%):		
1 (least deprived)	3712 (8%)	55,612 (9.3)
2	5510 (14%)	81,311 (13.5)
3	5273 (13%)	79,094 (13.2)
4	5329 (13%)	87,680 (14.6)
5 (most deprived)	5115 (13%)	77,959 (13.0)
Missing	16,014 (39.1)	219,727 (36.5)
Chronic obstructive pulmonary disease (%)	0	31,294 (5.2)
Hypertension (%)	27,783 (67.8)	401,918 (66.8)
Cardiovascular disease (%)	8090 (19.8)	169,805 (28.2)
Baseline smoking status (%)		
Nonsmoker	20,918 (55.7)	256,732 (49.2)
Ex-smoker	11,537 (30.7)	167,358 (32.1)
Current smoker	5129 (13.7)	98,001 (18.8)
Missing smoking status (%)	3369 (8.2)	79,292 (13.2)
ACEI type (%)		
Ramipril	22,600 (55.2)	324,942 (54.0)
Lisinopril	10,279 (25.1)	148,389 (24.7)
Perindopril	5741 (14.0)	91,054 (15.1)
Enalapril	1907 (4.7)	28,760 (4.8)
Other∗	426 (1.0)	8238 (1.4)
Discontinuing ACEIs, n (%)	18,973 (46.3)	271,773 (45.2)
Switching to angiotensin-II receptor blocker, n (%)	7108 (17.4)	88,980 (14.8)
Mean ACEI dose, mg (SD)†	4.4 (2.9)	4.5 (3.0)
Mean general practitioner consultations, n (SD)‡	12.4 (21.1)	12.0 (18.9)

*ACEI*, angiotensin converting enzyme inhibitor; *SD*, standardized difference.

∗ Other includes quinapril, trandolapril, captopril, fosinopril, imidapril, cilazapril, or moexipril.

† Standardized ramipril equivalent dose before switching.

‡ Mean number of general practice surgery consultations between the date of ACEI initiation and angiotensin-II receptor blocker initiation. *P* < .05 for all comparisons using chi-square test for counts and *t* test for continuous variables.

The HR for switching to an ARB in patients with active asthma was increased compared with the general population (HR = 1.16; 95% confidence interval [CI], 1.14-1.18) (Table II). In contrast, it was decreased for patients with COPD (HR = 0.89; 95% CI, 0.87-0.91). When associations among other patient characteristics were examined, the hazard of switching to an ARB was greater in women compared with men (HR = 1.46; 95% CI, 1.45-1.47), with increasing age (HR = 1.65; 95% CI, 1.62-1.71 for patients aged ≥60 years), and in patients with a BMI of 25 or greater (Table II). In contrast, the hazard of switching to an ARB was lower in patients with a history of smoking and in those registered at general practices in more socioeconomically deprived areas.

**Table II:** Hazard ratios (HRs) for switching to an angiotensin-II receptor blocker after any angiotensin converting enzyme inhibitor therapy in people with active asthma compared with general population and other risk factors∗

Characteristic	Crude HR (95% CI)	Crude *P*	Adjusted HR (95% CI)	Adjusted *P*
Population				
General population	1.00		1.00	
Active asthma	1.22 (1.20-1.24)	<.001	1.16 (1.14-1.18)	<.001
Chronic obstructive pulmonary disease	0.79 (0.78-0.81)	<.001	0.89 (0.87-0.91)	<.001
Hypertension	1.34 (1.33-1.35)	<.001	1.21 (1.20-1.22)	<.001
Cardiovascular disease	0.81 (0.80-0.82)	<.001	0.88 (0.87-0.89)	<.001
Sex				
Male	1.00		1.00	
Female	1.53 (1.52-1.54)	<.001	1.46 (1.45-1.47)	<.001
Age at baseline, y				
<40	1.00		1.00	
40-49	1.34 (1.30-1.37)	<.001	1.32 (1.29-1.36)	<.001
50-59	1.53 (1.50-1.57)	<.001	1.53 (1.49-1.57)	<.001
>60	1.67 (1.63-1.71)	<.001	1.66 (1.62-1.70)	<.001
Body mass index category				
<20	1.00		1.00	
20-24	1.37 (1.34-1.40)	<.001	1.43 (1.39-1.46)	<.001
≥25	1.52 (1.49-1.56)	<.001	1.55 (1.51-1.59)	<.001
Smoking status				
Nonsmoker	1.00		1.00	
Ex-smoker	0.89 (0.88-0.90)	<.001	0.96 (0.95-0.97)	<.001
Current smoker	0.64 (0.63-0.65)	<.001	0.73 (0.72-0.74)	<.001
Deprivation†				
1 (Least deprived)	1.00		1.00	
2	1.07 (1.05-1.08)	<.001	1.05 (1.04-1.06)	<.001
3	1.13 (1.12-1.14)	<.001	1.10 (1.09-1.11)	<.001
4	1.17 (1.15-1.18)	<.001	1.13 (1.12-1.15)	<.001
5 (Most deprived)	1.24 (1.22-1.25)	<.001	1.20 (1.18-1.21)	<.001

*CI*, confidence interval.

∗ Model was adjusted for gender, age, body mass index, smoking status, history of hypertension, cardiovascular disease, chronic obstructive pulmonary disease, and socioeconomic deprivation.

† Deprivation indicates index of multiple deprivation.

**Figure 1:**
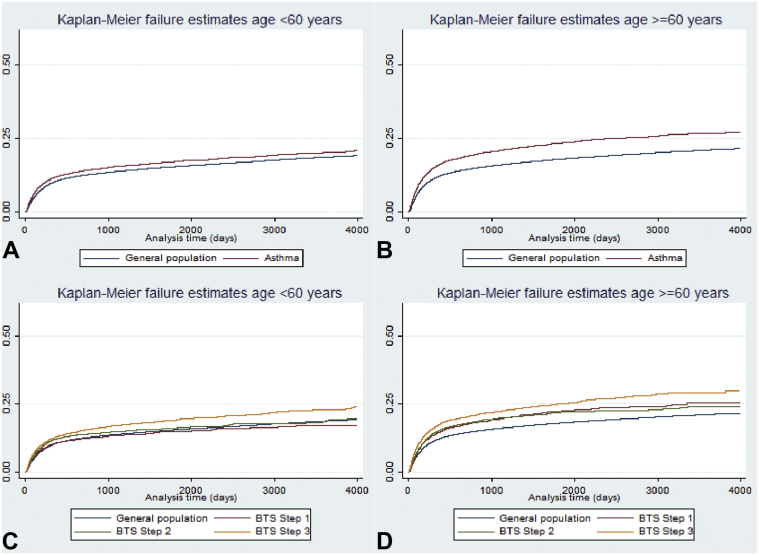
Kaplan-Meier failure plots for risk for switching to an angiotensin-II receptor blocker after treatment with angiotensin converting enzyme inhibitor in (**A**) people aged less than 60 years with asthma, (**B**) people aged less than 60 years by British Thoracic Society (BTS) treatment step, (**C**) people aged 60 years or older with asthma, and (**D**) people aged 60 years or older by BTS treatment step.

The increased hazard of switching to an ARB with active asthma was similar when stratified by sex (HR = 1.16, 95% CI, 1.13-1.19 for men; and HR = 1.17, 95% CI, 1.15-1.20 for women). Hazard ratios for switching to an ARB were greater among active asthma patients aged 60 years or greater and among those at BTS step 3 or greater (HR = 1.35, 95% CI, 1.32-1.39; and HR = 1.18, 95% CI, 1.15-1.22 for patients aged ≥60 and <60 years, respectively) (Figure 1 and Table III). Whereas the HR was elevated among asthma patients aged 60 years or greater at BTS steps 1 and 2, no increased hazard was observed for those aged less than 60 years. When stratified by the four most commonly prescribed ACEIs, the HR for switching to an ARB in patients with active asthma was consistently elevated for all ACEI types. It was numerically largest with enalapril (HR = 1.24; 95% CI, 1.17-1.32) (Table IV) and greatest in those at BTS step 3 or greater. Results of the sensitivity analysis using a complete case analysis were in keeping with the main results (see Table E3 in this article's Online Repository at www.jaci-inpractice.org).

**Table III:** Overall adjusted cause-specific hazard ratios (HRs) for switching to an angiotensin-II receptor blocker after angiotensin converting enzyme inhibitor therapy, stratified by British Thoracic Society asthma treatment step∗

British Thoracic Society asthma treatment step	With asthma, n (%)	Crude HR (95% CI)	Crude *P*	Adjusted HR (95% CI)	Adjusted *P*
Age ≥60 y					
≥3	9057 (45.6)	1.47 (1.44-1.51)	<.001	1.35 (1.32-1.39)	<.001
2	5774 (29.1)	1.22 (1.18-1.26)	<.001	1.13 (1.09-1.17)	<.001
1	5026 (25.3)	1.23 (1.19-1.28)	<.001	1.14 (1.09-1.19)	<.001
Age <60 y					
≥3	9398 (44.6)	1.27 (1.23-1.30)	<.001	1.18 (1.15-1.22)	<.001
2	4982 (23.6)	1.09 (1.05-1.14)	<.001	1.02 (0.96-1.07)	.753
1	6716 (31.8)	0.97 (0.94-1.01)	.193	0.96 (0.92-1.00)	.146

*CI*, confidence interval.

∗ Model was adjusted for sex, age, body mass index, smoking status, history of hypertension, history of cardiovascular disease, chronic obstructive pulmonary disease, and socioeconomic deprivation.

**Table IV:** Overall adjusted cause-specific hazard ratios (HRs) for switching to an angiotensin-II receptor blocker after different types of angiotensin converting enzyme inhibitor therapy∗

Angiotensin converting enzyme inhibitor type	Crude HR (95% CI)	Crude *P*	Adjusted HR (95% CI)	Adjusted *P*
Enalapril				
BTS step ≥3	1.51 (1.39-1.64)	<.001	1.44 (1.32-1.58)	<.001
BTS step 2	1.29 (1.16-1.42)	<.001	1.21 (1.08-1.35)	<.001
BTS step 1	1.04 (0.92-1.17)	.582	1.01 (0.89-1.16)	.841
Overall	1.31 (1.24-1.39)	<.001	1.25 (1.18-1.34)	<.001
Ramipril				
BTS step ≥3	1.34 (1.30-1.37)	<.001	1.27 (1.23-1.30)	<.001
BTS step 2	1.16 (1.12-1.20)	<.001	1.09 (1.05-1.14)	<.001
BTS step 1	1.05 (1.01-1.09)	.010	1.04 (1.00-1.08)	.060
Overall	1.21 (1.19-1.24)	<.001	1.16 (1.14-1.19)	<.001
Lisinopril				
BTS step ≥3	1.32 (1.27-1.37)	<.001	1.26 (1.21-1.31)	<.001
BTS step 2	1.14 (1.08-1.19)	<.001	1.09 (1.04-1.15)	.001
BTS step 1	1.10 (1.04-1.16)	<.001	1.10 (1.05-1.17)	<.001
Overall	1.21 (1.18-1.24)	<.001	1.17 (1.14-1.21)	<.001
Perindopril				
BTS step ≥3	1.36 (1.30-1.43)	<.001	1.27 (1.21-1.33)	<.001
BTS step 2	1.09 (1.01-1.17)	.026	1.03 (0.95-1.11)	.456
BTS step 1	1.01 (0.93-1.09)	.856	0.97 (0.89-1.05)	.410
Overall	1.20 (1.16-1.25)	<.001	1.13 (1.09-1.18)	<.001

*BTS step*, British Thoracic Society asthma treatment step; *CI*, confidence interval.

∗ Model was adjusted for sex, age, body mass index, smoking status, history of hypertension, cardiovascular disease, chronic obstructive pulmonary disease, and socioeconomic deprivation.

The overall incidence of switching to an ARB in the general population was 148/1000 patients, with an additional 24/1000 patients (95% CI, 21-27) among people with active asthma. The NNT with an ACEI for one person to switch to an ARB varied by age, sex, BMI, and asthma severity (Table V). The NNT in men with a BMI less than 20 varied from 24 to 11; it was lower with older patients at BTS step 3. Corresponding numbers for men with a BMI of 25 or greater were lower, ranging from 12 to 6, respectively. The NNT similarly varied in women, ranging from 14 to 7 in women with a BMI less than 20 and from 10 to 4 in women with a BMI of 25 or greater; it was lower in older patients at BTS step 3. Corresponding numbers for the general population are shown in Table E4 (in this article's Online Repository at www.jaci-inpractice.org).

**Table V:** Number of asthma patients needed to treat (NNT) with an angiotensin converting enzyme inhibitor for one person to switch to an angiotensin-II receptor blocker according to age, sex, body mass index, and asthma severity∗

Characteristic	Men				Women			
	Rate in non-asthmatics per 1000	NNT step 1 asthma	NNT step 2 asthma	NNT step ≥3 s-asthma	Rate in non-asthmatics per 1000	NNT step 1 asthma	NNT step 2 asthma	NNT step ≥3 asthma
BMI <20								
Age <40 y	9	24	24	21	74	14	14	11
Age 40-59 y	63	16	16	14	126	8	8	7
Age ≥60 y	68	13	13	11	114	8	8	7
BMI 20-24								
Age <40 y	63	16	16	14	99	10	10	9
Age 40-59 y	91	11	11	9	149	7	7	6
Age ≥60 y	114	8	8	7	176	5	5	4
BMI ≥25								
Age <40 y	82	12	12	10	101	10	10	8
Age 40-59 y	118	9	9	7	171	6	6	5
Age ≥60 y	135	7	7	6	192	5	5	4

*BMI*, body mass index (kg/m^2^); *step*, British Thoracic Society asthma treatment step.

∗ Rate indicates the rate of switching to an angiotensin-II receptor blocker after angiotensin converting enzyme inhibitor initiation. The NNT was calculated taking the reciprocal of the rate in the non-asthma population times the hazard ratio of switching in asthma by age and British Thoracic Society step, rounded to the nearest whole number.

## Discussion

### Summary of findings

We observed that people with active asthma have an increased risk for ACEI intolerance and switching to ARB therapy compared with the general population. This association was greatest in those with more severe asthma. People age greater than and less than 60 years at BTS step 3 or greater asthma have a 35% increased hazard versus 18% increased hazard, respectively. The hazard of switching to an ARB was consistently elevated with all commonly prescribed ACEIs in this population and was largest after treatment with enalapril; BTS step 3 or greater patients had a 44% increased hazard. However, patients age less than 60 years at BTS step 1 or 2 asthma were not at increased risk. The number of asthma patients NNT with ACEI for one person to switch was also significantly influenced by age, sex, and BMI, which ranged from 21 to 4; it was lowest in older women with a BMI of 25 or greater at BTS step 3.

### Comparison with previous literature

Airway hyperresponsiveness is an important determinant in the pathophysiology of asthma and is affected by a variety of stimuli such as methacholine and bradykinin, which can cause bronchoconstriction.^7^^,^^8^ Whereas methacholine induces bronchoconstriction in normal and in asthmatic subjects, bradykinin-induced bronchoconstriction is predominantly observed in asthmatic patients, which suggests that the effect of bradykinin is related to structural and/or functional airway abnormalities that occur in asthma.^7^ Bradykinin's potent bronchoconstrictor effect in asthmatic patients is thought to be mediated through an indirect mechanism related to the level of AHR and active airway inflammation.^9^^,^^10^ Whereas the increased hazard of switching in people with active asthma, but not COPD, would be in keeping with a specific effect on AHR, other mechanisms, such as ACEI increasing cough reflex hypersensitivity, which is similarly associated with female sex, cannot be excluded.^21^

Indirect acting AHR is related to the degree of aeroallergen sensitization and occurs independently of airway caliber or ICS use.^22^ This in turn may explain why the effect of bradykinin resulting from ACEI may be specific for asthma but not COPD, in addition to the presence of type 2 inflammation in the former. This is because AHR is not a key feature in the pathogenesis of COPD, perhaps unless patients have asthma–COPD overlap syndrome. Indeed, fixed airway remodeling in COPD may be one reason why a decreased hazard of switching was observed in this population. Our observation of increased ACEI intolerance in patients with BTS step 3 and above may be because such patients have more severe disease. Nevertheless, AHR has been shown to be attenuated by drugs such as ICS, which would be more prevalent in patients taking step 3/4 therapy.^23^, ^24^, ^25^ Some studies evaluated bronchial reactivity of captopril, ramipril, and enalapril in asthma patients and showed no change in reactivity.^26^, ^27^, ^28^, ^29^, ^30^, ^31^ However, the cumulative number of patients from all of those studies is only n = 71, which, in addition to studies employing different methods (ie, histamine, bradykinin, or methacholine challenges or simply measuring lung function), limits the generalizability of the findings.

Although several types of ACEIs are available for clinical use, it cannot be assumed that they are all equally effective or safe without head-to-head comparisons. In our study, the hazard of switching to ARB with enalapril was modestly larger in people with asthma compared with other ACEIs. In a meta-analysis of randomized controlled trials, ACEI cough had higher rates in hypertension and lowest rates in heart failure, which suggests that these may differ by underlying cardiovascular condition.^32^ Although differences among users of different ACEI types remain possible, we adjusted for several of these factors and saw a larger HR for hypertension compared with cardiovascular disease. Similarly, a network meta-analysis of 29 randomized placebo-controlled trials of ACEI therapy in heart failure patients found that enalapril had the highest incidence of cough, gastrointestinal discomfort, and greater deterioration in renal function compared with other ACEIs.^33^

An increased risk for cough or switching to ARB therapy in people with asthma was recently reported.^32^^,^^34^ However, no studies used an active asthma population, examined associations by asthma severity or type of ACEI, or provided information relating to ACEI dose or the rate of health care use before switching. Moreover, information on absolute risk is lacking but is necessary to guide robust health economic and clinical decision-making. Women in the general population are considered to have a 1.5- to 2.3-fold increased risk for switching to ARBs after ACEI therapy.^35^, ^36^, ^37^ However, the impact of increasing age has been less consistently reported and a paucity of data remains regarding the association with BMI.^38^, ^39^, ^40^ We clearly show that all three characteristics are relevant for people with asthma and are strong determinants of the NNT.

### Strengths and limitations

This study had several strengths and limitations. First, we analyzed a large clinical population identified using a validated data source and definitions. Although cough is by far the most common reason for ACEI intolerance and switching to an ARB, we were unable to measure ACEI-induced cough directly as an outcome. This would be challenging, because cough may not be recorded sufficiently to distinguish between cough related to ACEIs as opposed to another condition, particularly in patients with asthma. Although cough is the predominant reason for ACEI intolerance in the general population, we cannot exclude the possibility that other symptoms such as wheeze or dyspnea may have occurred, which have been reported among asthma patients using ACEIs.^40^ However, switching to an ARB after ACEI treatment is considered to be the best marker for identifying ACEI-induced adverse drug reactions in electronic databases. This has a positive predictive value of up to 90.5%, in which cough is the most commonly reported adverse reaction.^41^^,^^42^

The potential remains for unmeasured confounding from potentially important unknown patient factors not included in this model, but we used a negative control population by examining the association in patients with COPD. The null findings in patients with COPD provide additional evidence suggesting that our observed association is causal and that the increased hazard of switching observed in people with active asthma is potentially related to changes in AHR owing to bradykinin. However, these results may not be generalizable to people with asthma–COPD overlap syndrome. It would be pertinent to evaluate the putative impact of ACEI further in patients with known AHR and markers of type 2 inflammation, such as fractional exhaled nitric oxide and blood eosinophils, as well as total and specific IgE levels.^43^^,^^44^

### Clinical implications

It is recognized that managing comorbidities in patients with asthma may be associated with additional risk.^45^, ^46^, ^47^, ^48^ When evaluated for the management of hypertension, ARBs are thought to have similar effects on blood pressure, mortality, and cardiovascular disease outcomes compared with ACEIs, yet fewer patients in the general population withdraw from clinical trials because of adverse effects when treated with ARBs compared with ACEIs.^49^ Despite the potentially higher incidence of switching with enalapril, the largest determinant regarding absolute risk in people with asthma appeared to be a person's age, sex, and BMI. Given the high prevalence of obesity in the population combined with the increasing age of patients, such factors are important determinants for considering whether ARBs should be recommended as first-line therapy. This would be particularly relevant in people with asthma, for whom discriminating ACEI-induced cough from symptoms of uncontrolled asthma may be complex, potentially leading to unnecessary asthma treatment if not immediately recognized. Many guidelines for the management of patients with cardiovascular disease continue to recommend ACEIs as first-choice therapy, reserving ARBs as an alternative when patients are intolerant to ACEIs. This has led to recent calls to change these recommendations because of the equal efficacy but fewer adverse reactions with ARBs.^50^ This would potentially avoid unnecessary health care appointments, patient treatment disutility, and delays in establishing effective therapy for the underlying clinical condition.

Our findings suggest that ACEIs are less well tolerated in people with asthma compared with the general population. The NNT is lower in asthma and in those who are older age and female, and who have a higher BMI. Consideration could potentially be given to recommending ARBs first in people with asthma or those with high-risk characteristics when treatment with a renin-angiotensin system inhibitor is clinically indicated.

## Figures and Tables

**Table E1:** Method of calculating ramipril equivalent doses

Angiotensin converting enzyme inhibitor	Conversion to ramipril equivalent strength
Captopril	Captopril strength/10
Cilazapril	Cilazapril strength/1
Enalapril	Enalapril strength/2
Fosinopril	Fosinopril strength/4
Imidapril	Imidapril strength/2
Lisinopril	Lisinopril strength/2
Perindopril	Perindopril strength × 1.25
Trandolapril	Trandolapril strength × 2.5

**Table E2:** Codes used to identify angiotensin converting enzyme inhibitor and angiotensin-II receptor blocker prescriptions in Clinical Practice Research Datalink (CPRD)

CPRD angiotensin converting enzyme inhibitor product codes
65, 69, 78, 80, 82, 97, 147, 196, 217, 277, 448, 593, 633, 654, 709, 756, 761, 1021, 1121, 1143, 1144, 1299, 1520, 107, 1904, 2927, 2982, 3069, 3203, 3310, 3509, 3720, 3839, 3929, 4103, 4571, 5047, 5159, 5189, 5275, 5612, 573, 5800, 5861, 6078, 6200, 6261, 6288, 6314, 6359, 6362, 6364, 6408, 6468, 6765, 6786, 6794, 6806, 6807, 7314, 7419, 8025, 8026, 8105, 8106, 8268, 8800, 8830, 8923, 9646, 9693, 9731, 9764, 9915, 9948, 10882, 10902, 1113, 11197, 11351, 11561, 11567, 11641, 11937, 11965, 11983, 11987, 12313, 12411, 12412, 12574, 12815, 1288, 13026, 13589, 13755, 14228, 14387, 14477, 14478, 14960, 15031, 15085, 15096, 15108, 15121, 15135, 1565, 15958, 16196, 16197, 16212, 16701, 16708, 16710, 16924, 17006, 17120, 17474, 17624, 17633, 17655, 1829, 18223, 18263, 18269, 18325, 19198, 19204, 19208, 19223, 19690, 20188, 20579, 20849, 20975, 21053, 2112, 21231, 21943, 22004, 22439, 22708, 2882, 23252, 23382, 23478, 23642, 24041, 24214, 24482, 24693, 2505, 25998, 26995, 27871, 27890, 28127, 28438, 28486, 28586, 28724, 28725, 28820, 28902, 29130, 29530, 29627, 29964, 30039, 30921, 31288, 31307, 31587, 31716, 31810, 32048, 32166, 32241, 32514, 32560, 32597, 32857, 32934, 33057, 33078, 33095, 33336, 33353, 33646, 33811, 33894, 33977, 34357, 34382, 34390, 34400, 34412, 34429, 34431, 34432, 34453, 34471, 34490, 34505, 34528, 34539, 34540, 34544, 34562, 34567, 34583, 34589, 34651, 34652, 34657, 34696, 34698, 34710, 34712, 34719, 34732, 34768, 34798, 34799, 34877, 34893, 34936, 34937, 34943, 34952, 34953, 35007, 35302, 35731, 35794, 36742, 36753, 37080, 37087, 37655, 37710, 37778, 37908, 37930, 37964, 37965, 37971, 37978, 38026, 38034, 38285, 38308, 38510, 38854, 38899, 38995, 39137, 39147, 39227, 39242, 39355, 39421, 39512, 40355, 40384, 41417, 41522, 41532, 41538, 41573, 41617, 41633, 41694, 41743, 41746, 42081, 42285, 42723, 42894, 42901, 42902, 42908, 43012, 43411, 43412, 43413, 43416, 43418, 43432, 43507, 43563, 43566, 43649, 43813, 44527, 44657, 45217, 45228, 45264, 45300, 45319, 45324, 45337, 45340, 45554, 45816, 45938, 46365, 46851, 46890, 46951, 46957, 46974, 46975, 46979, 47021, 47159, 47998, 48008, 48049, 48053, 48098, 48180, 48214, 49164, 49491, 50334, 50347, 50402, 50509, 50607, 50780, 50863, 51258, 51433, 51701, 51714, 51807, 52010, 52088, 52197, 52293, 52399, 52407, 52499, 52882, 53058, 53271, 53551, 53612, 53621, 53719, 53820, 53915, 54037, 54201, 54283, 54288, 54298, 54345, 54512, 54544, 54620, 54733, 54899, 54928, 54941, 54942, 54986, 55002, 55299, 55399, 55456, 55588, 55639, 55798, 55896, 55903, 56013, 56038, 56079, 56129, 56148, 56157, 56162, 56169, 56244, 56279, 56356, 56472, 56473, 56505, 56506, 56508, 56509, 56510, 56516, 56704, 56763, 56850, 56855, 57048, 57073, 57235, 57333, 57346, 57378, 57539, 57588, 57658, 57701, 57801, 57864, 57882, 57944, 58195, 58258, 58294, 58451, 58461, 58682, 58751, 58843, 58863, 58871, 58874, 59109, 59111, 59557, 59603, 59699, 59770, 59788, 59790, 59915, 59972, 59996, 60010, 60065, 60067, 60097, 60143, 60232, 60309, 60349
CPRD angiotensin-II receptor blocker product codes
51368, 51897, 56606, 529, 531, 4155, 4685, 4741, 4818, 5013, 5117, 7043, 31072, 50185, 51117, 51519, 51647, 52208, 52559, 53680, 53755, 54326, 54414, 57026, 57266, 57273, 57977, 58646, 59690, 59802, 6939, 9745, 1836, 13123, 16285, 16371, 10316, 11448, 35196, 828, 1293, 2971, 7338, 9196, 11348, 36939, 52972, 55017, 5808, 58201, 59393, 60597, 31160, 11469, 11526, 35481, 37650, 52658, 59903, 520, 624, 1780, 4226, 5723, 1473, 14965, 24632, 38367, 39944, 40571, 40711, 41232, 47006, 48398, 49492, 49588, 50971, 51186, 51601, 5242, 52659, 52886, 54049, 54057, 54404, 54735, 54740, 54843, 55296, 55446, 55718, 56104, 56970, 57028, 58274, 58649, 58967, 59086, 59271, 59340, 59351, 59750, 60506, 4540, 6437, 10323, 21423, 37747, 48039, 52189, 5160, 56204, 56975, 57796, 46687, 46715, 46792, 47467, 55358, 47573, 35380, 39021, 46355, 53220, 47616, 4727, 18903, 43322, 43915, 6217, 6285, 6351, 14983, 18910, 20117, 39786, 39984, 40316, 40639, 40668, 4120, 41205, 18200, 27520, 29634, 60007, 38459, 38889, 5988, 6243, 12874, 13821, 17545, 17686, 14870, 16161, 1689, 18202, 35173, 35174, 35304, 35317, 764, 16060, 52858, 575, 3222, 4645, 6518, 11251, 11252, 14943, 2346, 24268, 24359, 24484, 37573, 38395, 39199, 44778, 45600, 53833, 55187, 58669, 58910, 59029, 59448, 6006, 54726, 55821, 35096, 35189, 35329, 35343, 35697, 6877, 11864, 14283, 25382

**Table E3:** Complete case analysis of switching to an angiotensin-II receptor blocker after angiotensin converting enzyme inhibitor therapy in people with active asthma compared with the general population and other risk factors

Characteristic	Crude hazard ratio (95% confidence interval)	Crude *P*	Adjusted hazard ratio (95% confidence interval)	Adjusted *P*
Population				
General population	1.00		1.00	
Active asthma	1.22 (1.19-1.25)	<.001	1.16 (1.13-1.20)	<.001
Chronic obstructive pulmonary disease	0.83 (0.80-0.86)	<.001	0.93 (0.89-0.98)	.002
Hypertension	1.30 (1.28-1.32)	<.001	1.14 (1.12–1.16)	<.001
Cardiovascular disease	0.83 (0.82-0.85)	<.001	0.92 (0.90-0.94)	<.001
Sex				
Male	1.00		1.00	
Female	1.56 (1.54-1.58)	<.001	1.52 (1.50-1.55)	<.001
Age at baseline, y				
<40	1.00		1.00	
40-49	1.33 (1.27-1.39)	<.001	1.32 (1.24-1.40)	<.001
50-59	1.53 (1.47-1.60)	<.001	1.55 (1.46-1.64)	<.001
>60	1.72 (1.65-1.79)	<.001	1.73 (1.64-1.83)	<.001
Body mass index category				
<20	1.00		1.00	
20-24	1.33 (1.27-1.39)	<.001	1.37 (1.29-1.45)	<.001
≥25	1.43 (1.37-1.50)	<.001	1.54 (1.45-1.63)	<.001
Smoking status				
Nonsmoker	1.00		1.00	
Ex-smoker	0.90 (0.89-0.92)	<.001	0.98 (0.96–0.999)	.045
Current smoker	0.61 (0.60-0.63)	<.001	0.70 (0.68–0.72)	<.001
Deprivation				
1 (least deprived)	1.00		1.00	
2	1.09 (1.07-1.12)	<.001	1.07 (1.04-1.10)	<.001
3	1.11 (1.09-1.15)	<.001	1.07 (1.04-1.10)	<.001
4	1.17 (1.15-1.17)	<.001	1.10 (1.07-1.13)	<.001
5 (most deprived)	1.24 (1.21-1.27)	<.001	1.20 (1.16-1.23)	<.001

**Table E4:** Complete case analysis measuring association between asthma British Thoracic Society (BTS) treatment step and switching to an angiotensin-II receptor blocker, according to age

Characteristic	Crude hazard ratio (95% confidence interval)	Crude *P*	Adjusted hazard ratio (95% confidence interval)	Adjusted *P*
Age ≥60 y				
BTS step ≥3	1.49 (1.42-1.56)	<.001	1.37 (1.29-1.46)	<.001
BTS step 2	1.22 (1.15-1.30)	<.001	1.10 (1.01-1.19)	.025
BTS step 1	1.21 (1.14-1.29)	<.001	1.07 (0.99-1.17)	.104
Age <60 y				
BTS step ≥3	1.30 (1.24-1.37)	<.001	1.24 (1.16-1.32)	<.001
BTS step 2	1.08 (1.01-1.17)	.035	1.01 (0.91-1.12)	.870
BTS step 1	1.02 (0.95-1.09)	.602	1.02 (0.94-1.12)	.594

**Table E5:** Absolute risk for switching to an angiotensin-II receptor blocker after treatment with angiotensin converting enzyme inhibitors in the general population, stratified by age, sex, and body mass index (BMI)

Characteristic	General population			
	Men		Women	
	Rate/1000	NNT overall	Rate/1000	NNT overall
BMI <20				
Age <40 y	41	24	74	14
Age 40-49 y	57	18	140	7
Age 50-59 y	69	14	112	9
Age >60 y	68	15	114	9
BMI 20-24	0		0	
Age <40 y	63	16	99	10
Age 40-49 y	81	12	133	8
Age 50-59 y	101	10	165	6
Age >60 y	114	9	176	6
BMI ≥25	0		0	
Age <40 y	82	12	101	10
Age 40-49 y	107	9	157	6
Age 50-59 y	129	8	184	5
Age >60 y	135	7	192	5

*NNT*, number needed to treat with an angiotensin converting enzyme inhibitor for one person to switch to an angiotensin-II receptor blocker.
